# Childhood Stroke: Awareness, Interest, and Knowledge Among the Pediatric Community

**DOI:** 10.3389/fped.2018.00182

**Published:** 2018-06-25

**Authors:** Michaela V. Bonfert, Katharina Badura, Julia Gerstl, Ingo Borggraefe, Florian Heinen, Sebastian Schroeder, Martin Olivieri, Raphael Weinberger, Mirjam N. Landgraf, Katharina Vill, Moritz Tacke, Steffen Berweck, Karl Reiter, Florian Hoffmann, Thomas Nicolai, Lucia Gerstl

**Affiliations:** ^1^Department of Pediatric Neurology and Developmental Medicine, Dr. von Hauner Children's Hospital, University Hospital, Ludwig Maximilians University of Munich, Munich, Germany; ^2^Schön Klinik Vogtareuth, Clinic for Neuropediatrics and Neurorehabilitation, Epilepsy Center for Children and Adolescents, Vogtareuth, Germany; ^3^Department of Pediatrics, Facharztzentrum Hauzenberg, Hauzenberg, Germany; ^4^Department of Pediatric Hemostaseology, Dr. von Hauner Children's Hospital, University Hospital, Ludwig Maximilians University of Munich, Munich, Germany; ^5^Pediatric Intensive Care Unit, Dr. von Hauner Children's Hospital, University Hospital, Ludwig Maximilians University of Munich, Munich, Germany; ^6^Division of Epidemiology, Institute of Social Pediatrics and Adolescent Medicine, University Hospital, Ludwig Maximilians University of Munich, Munich, Germany

**Keywords:** childhood stroke, pediatric stroke, awareness, face-arm-speech-test, stroke mimics, stroke symptoms, stroke therapy, stroke diagnostics

## Abstract

**Objective:** Acute childhood stroke is an emergency requiring a high level of awareness among first-line healthcare providers. This survey serves as an indicator of the awareness of, the interest in, and knowledge of childhood stroke of German pediatricians.

**Methods:** Thousand six hundred and ninety-seven physicians of pediatric in- and outpatient facilities in Bavaria, Germany, were invited via email to an online-survey about childhood stroke.

**Results:** The overall participation rate was 14%. Forty-six percent of participants considered a diagnosis of childhood stroke at least once during the past year, and 47% provide care for patients who have suffered childhood stroke. The acronym FAST (Face-Arm-Speech-Time-Test) was correctly cited in 27% of the questionnaires. Most commonly quoted symptoms of childhood stroke were hemiparesis (90%), speech disorder (58%), seizure (44%), headache (40%), and impaired consciousness (33%). Migraine (63%), seizure (39%), and infections of the brain (31%) were most frequently named as stroke mimics. Main diagnostic measures indicated were magnetic resonance imaging (MRI) (96%) and computer tomography (CT) (55%). Main therapeutic strategies were thrombolysis (80%), anticoagulation (41%), neuroprotective measures, and thrombectomies (15% each). Thirty-nine percent of participants had taken part in training sessions, 61% studied literature, 37% discussed with colleagues, and 25% performed internet research on childhood stroke. Ninety-three percent of participants approve skill enhancement, favoring training sessions (80%), publications (43%), and web based offers (35%). Consent for offering a flyer on the topic to caregivers in facilities was given in 49%.

**Conclusion:** Childhood stroke constitutes a topic of clinical importance to pediatricians. Participants demonstrate a considerable level of comprehension concerning the subject, but room for improvement remains. A multi-modal approach encompassing an elaborate training program, regular educational publications in professional journals, and web based offers could reach a broad range of health care providers. Paired with a public adult and childhood stroke awareness campaign, these efforts could contribute to optimize the care for children suffering from stroke.

## Introduction

Given the reported annual incidence of pediatric arterial ischemic and hemorrhagic stroke of 1.2–13/100,000, strokes belong to the rare conditions in the pediatric population (1). However, an acute stroke is a (neuro-)pediatric emergency and has a high mortality rate (10%) as well as a substantial impact on quality of life of survivors and their families. Consequences of a stroke, including the impairment of hand-use, mobility, and behavioral or academic performance hinder development crucial for independent living, participation in everyday life, and quality of life in a significant number of patients (2). Subsequently, a substantial economic burden results on the health care system as well as for the families (3, 4). Best clinical outcomes after a childhood stroke are only possible with rapid access to adequate neuroprotective intensive care, as well as to hyperacute management strategies proven effective in adults (5, 6). However, the diagnosis of acute stroke is delayed in a considerable number of children. Several aspects contribute to this delay: (1) low overall incidence, (2) heterogeneity of etiology and risk factors, (3) the non-specificity and the wide range of (age-dependent) symptoms, (4) the general challenge of recognizing mild focal signs, particularly in infants, (5) the broad differential diagnosis comprising more common pediatric disorders, such as migraine, seizure, or Bell's palsy, and (6) process factors, based on the availability of neuroimaging modalities. Furthermore, given the low a priori probability and the oftentimes puzzling clinical diagnosis, the rare suspicion of childhood stroke among health care providers is assumed to be one of the key issues in delayed diagnoses (7–12). However, low recognition rates could become subject to change if overall knowledge of the disorder improves, as has been shown for adult stroke (13–16). In this context, the necessity for interventions aimed at raising the professional awareness of childhood stroke becomes evident (17, 18). However, prior to the successful implementation of such measures, a thorough demand analysis is essential. To our knowledge, no published trial has systematically assessed the professional awareness and knowledge of childhood stroke, nor has any study addressed the commitment of health care providers to participate in interventions on this topic, or evaluated such programs thus far. The aim of this study was to get reliable information on (1) the rate of awareness of childhood stroke, (2) the level of knowledge that future education efforts may build on, and (3) the acceptance of different educational measures in a representative cohort of pediatricians. These results will facilitate the successful development and realization of a well-accepted and therefore cost-efficient training program on the topic of childhood stroke.

## Materials and methods

The survey was developed by experts in pediatric neurology, pediatric hemostaseology, and pediatric emergency medicine of our research group. The survey was embedded into the online tool *LimeSurvey* and piloted in between our research group for a period of 7 days to check for content clarity and technical soundness. The anonymous online-survey was finally launched March 23rd and conducted until May 22nd 2017. Thousand six hundred and ninety-seven participants were invited to complete the survey via email. After sending the invitation, the online-survey stayed accessible for a period of 2 months. Every 2 weeks a reminder message was sent.

### Survey

The survey included the following three sections: (1) introductive information, (2) a note regarding the protection of data privacy—this information on anonymous data-management had to be confirmed to begin completing the survey, and (3) the questionnaire itself. Participants were asked three questions on their professional background, two questions on the importance of childhood stroke in their daily professional routine, seven specific questions on the topic of childhood stroke itself [knowledge of the stroke screening tool Face-Arm-Speech-Time test (FAST), symptoms and differential diagnoses, diagnostics, and management of acute childhood stroke], three questions on their participation in former childhood stroke educational interventions and the former way physicians collected specific information on the topic respectively, and three questions on the demand for future stroke specific interventions from physicians' perspectives, and preferred methods of implementation. Question type differed depending on the topic, details can be found in the results section. The survey was administered in German, an English version is available in the Supplementary Material Section. The completion of the entire survey took ~5 min.

### Participants

Thousand two hundred and fifty-eight of the addressed 1,697 pediatricians are members of the Bavarian section of a German association of pediatricians practicing in doctor's offices. Furthermore, all pediatricians working at three children's hospitals or their social pediatric centers (SPZ) located in Munich, Germany (*n* = 391) and a neuropediatric rehabilitation center (*n* = 48) in the vicinity of Munich were asked to participate.

### Data management and statistics

All data entered by participants on the survey-platform were centrally recaptured. For further analysis, complete surveys were assessed. Plausibility checks, analysis, and figures were completed using Microsoft Excel.

### Ethical approval

The study was approved by the ethics committee and data protection commissioner of the medical faculty of the Ludwig-Maximilians-University Munich, Germany, Project-Nr. 455–16.

## Results

### Return rate

The online survey reached an overall participation rate of 14%. Two forty-five surveys were submitted, 53 were excluded resulting in a total of 192 complete surveys representing an 11% response rate. The following reasons led to exclusion: nine surveys were discontinued following the introductive information section (4%), and in eight surveys the note on the protection of data privacy was not agreed to (3%). Thirty-four surveys were recaptured without any of the questions been answered (14%), and two surveys without any information on the professional background, or further requirements of education on the topic (1%), respectively.

### Pediatric professional background (multiple-choice questions)

Questionnaires indicated participants practice in the following settings: 92 in a children's hospital (48%), 69 in a doctors' office (36%), 17 in a SPZ (9%), and 14 in a neuropediatric rehabilitation clinic (7%). Level of pediatric training was indicated as *residents in pediatrics* in 38 and *pediatricians* in 154 cases (80%). Fifty-eight pediatricians (30%) stated a further specialization in one of the following pediatric fields: 20 in *neurology* (35%), 13 in *neonatology* (22%), six in *intensive care* (10%), five in *cardiology* (9%), four in *hemostaseology* (7%), four in *pulmonology* (7%), two in *endocrinology* (3%), and one of each in *hematology/oncology, gastroenterology/hepatology, nephrology*, and *allergology*.

### Importance of childhood stroke in daily routines (multiple-choice questions)

**Figure 1:**
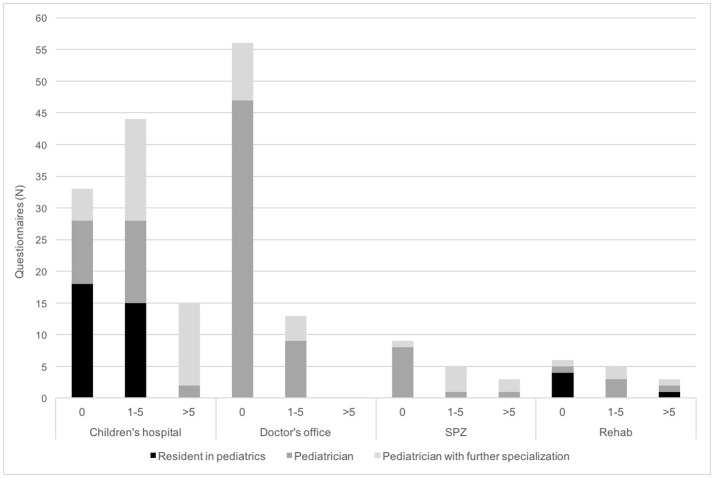
Importance of acute childhood stroke in daily routines of pediatricians. Physicians were asked for the number of patients in whom they considered childhood stroke during the past 12 months. N, number; SPZ, social pediatric center.

**Figure 2:**
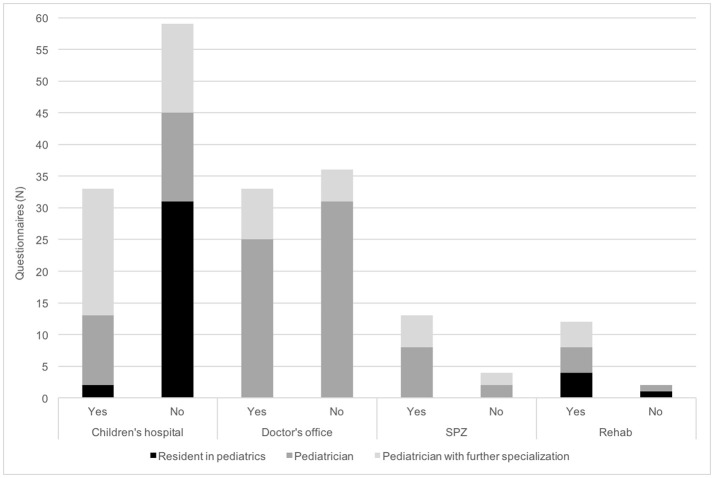
Importance of providing care for patients who have suffered childhood stroke in daily routines of pediatricians. Physicians were asked if they follow up patients who have suffered a childhood stroke. N, number; SPZ, social pediatric center.

Participants declared having considered childhood stroke in at least one patient during the past year in 88 questionnaires (46%). In 67 and 21 cases suspicion of childhood stroke arose 1–5 (35%) and more than 5 times (11%) during the past year, respectively (Figure 1). In 91 questionnaires participants confirmed providing care for patients who have suffered childhood stroke (47%) (Figure 2).

### Specific knowledge on childhood stroke

**Figure 3:**
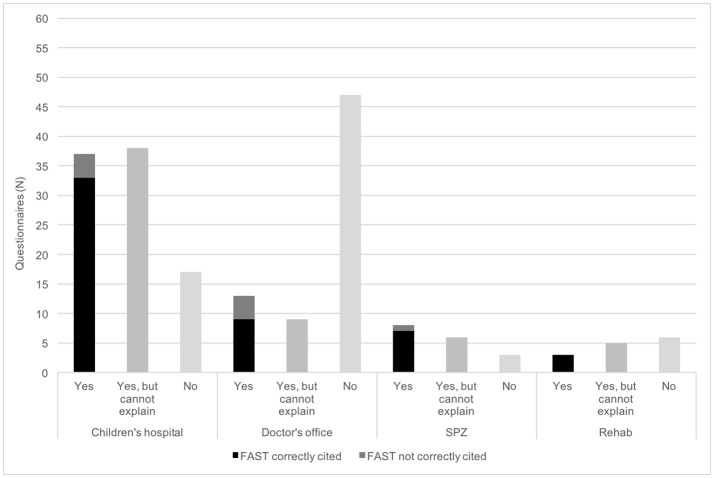
Knowledge of the mnemonic FAST (Face-Arm-Speech-Time) of pediatricians. Firstly, physicians were asked if they are used to the acronym FAST. Secondly, the meaning of the single letters should be cited. N, number; SPZ, social pediatric center.

FAST was confirmed to be a well-known acronym in the multiple-choice section of 61 questionnaires (32%), and its meaning was correctly cited in the free text field of 52 questionnaires (27%). Participants stated to have heard about FAST, but were unable to explain it in 58 cases (30%). In the further 73 questionnaires knowledge of FAST was denied (38%). FAST was properly specified by respondents practicing in SPZs, children's hospitals, neuropediatric rehabilitation facility and doctor's offices in 7 (41%), 33 (36%), 3 (21%), and 9 (13%) cases, respectively (Figure 3).

Asked for symptoms of childhood stroke, the most common free text answers included hemiparesis (*n* = *172*; 90%), speech disorder (*n* = *111; 58*%), seizure (*n* = *85; 44*%), headache (*n* = *77*; 40%), and impairment of consciousness (*n* = 64; 33%) (Tables 1, 2). Migraine (*n* = *120; 63*%), seizure/epilepsy (*n* = *75; 39*%), and infections of the brain (*n* = 59, 31%) were the most commonly cited disorders mimicking childhood stroke in the corresponding free text field (Tables 1, 2). In the context of diagnostic modalities for adequately confirming childhood stroke, the leading free text answer was magnetic resonance imaging (MRI) (+/− MR-angiography) (*n* = 185; 96%). Less frequently computer tomography (CT) (+/− CT-angiography) was stated (*n* = 105; 55%) followed by conventional angiography and transfontanellar ultrasound (*n* = *27; 14*% each) (Tables 1, 2). Thrombolysis (*n* = 154; 80%) was the most commonly proposed therapeutic strategy, followed by anticoagulation (*n* = *78; 41*%). Less frequently neuroprotective measures (*n* = *30; 15*%) and thrombectomies (*n* = *29; 15*%) were quoted in the free text fields, concerning acute management options of childhood stroke (Tables 1, 2). Maximum treatment time for these interventions was set to up to 6 h after onset of symptoms by the majority of responding physicians. Respondents were not required to answer all provided free text boxes to continue the questionnaire. The number of answers given in relation to the maximum answers possible yields to 87% in the questions of symptoms, 80% on mimics, 70% in diagnostics, and 60% on therapy (Table 3).

**Table 1:** Free text answers given by physicians to specific questions on the topic of pediatric stroke.

**Topic**	**Stroke symptoms**	***N***	**Stroke mimics**	***N***	**Diagnostic modality to confirm stroke diagnosis**	***N***	**Acute therapeutic options**	***N***
**Given free text answers**	Hemiparesis	172	Migraine	120	MRI (+/− MR angiography)	185	Thrombolysis	154
	Speech disorder	111	Seizure/epilepsy	75	CT (+/− CT angiography)	105	Anticoagulation	78
	Seizure	85	Brain infection	59	Conventional angiography	27	Neuroprotective measures	30
	Headache	77	Brain tumor	34	Transfontanellar Ultrasound	27	Intervention not further specified	29
	Impaired consciousness	64	Cerebral hemorrhage	22	Laboratory testing	20	Thrombectomy	29
	Disturbance of vision	30	Metabolic disorder	21	Clinical examination	18	Decompression	10
	Facial palsy	22	Autoinflammatory disease	16				
	(Focal) neurological deficit	14	Thrombosis	13				
	Vegetative symptoms	13	Facial palsy	12				
	Paresthesia	13	Hemiparesis	11				
	Dizziness	11	Borreliosis	10				
	Ataxia / difficulty walking	15	Somatoform disorder	10				
	Cranial nerve disorder	10	Traumatic brain injury	10				

*All answers given at least in 10 out of 192 analyzed questionnaires are shown. For further information on answers given less often refer to Table 2. Please list symptoms of childhood stroke; Please list mimics of childhood stroke; What kind of diagnostic modality contributes to confirm childhood stroke?; What kind of treatment options for acute intervention in case of childhood stroke do you know? N, number; MRI, magnetic resonance imaging; CT, computer tomography*.

**Table 2:** Free text answers given < 10 times in 192 questionnaires by physicians to specific questions on the topic of pediatric stroke.

**Topic**	**Stroke symptoms**	***N***	**Stroke mimics**	**N**	**Diagnostic modality to confirm stroke diagnosis**	**N**	**Acute therapeutic options**	**N**
Given free text answers	Apathy	1	Moya-Moya, Vasculitis, Vasculopathy	5	EEG	7	Functional therapy	4
	Apnoea	1	TIA	5	Doppler / Duplex	6	Epoetin	1
	Apraxia	1	Developmental speech delay	3	History	3	Medication	1
	Asymptomatic	1	Heart disease	3	Imaging	2	Treatment of underlying disorder	1
	Ataxia	1	Hypertension	2	Lumbar puncture	2		
	Drivel	1	PRES	2	Monitoring of blood pressure	2		
	Encephalopathy	1	Syncope	2	Contrast agent	1		
	Enuresis	1	Tension type headache	2				
	Like adults	1	Apathy	1				
	Meningism	1	Chassaignac paresis	1				
	Pain	1	Circulatory disorder	1				
	Retardation	1	Disorder of coagulation	1				
	Shrill crying	1	Insolation	1				
	Signs of ICP	1	Myopathy	1				
	Spasticity	1	Neurodegenrative disorder	1				
	Unbalancing	1	Retardation	1				
		1	Strabism	1				
		1	Sepsis	1				
		1	Sickle cell anemia	1				
		1	SID	1				
		1	Vertigo	1				

*The following questions were asked: Please list symptoms of childhood stroke; Please list mimics of childhood stroke; What kind of diagnostic modality contributes to confirm childhood stroke?; What kind of treatment options for acute intervention in case of childhood stroke do you know? N, number; TIA, transient ischemic attack; PRES, posterior reversible encephalopathy syndrome; SID, sudden infant death; EEG, electroencephalography*.

**Table 3:** Number of answers given by 192 physicians to free text questions concerning symptoms, mimics, diagnostics, and therapy of pediatric stroke.

**Topic**		**Symptoms**	**Mimics**	**Diagnostics**	**Therapy**
Free text boxes (*n*)		4	3	3	3
Total of answers (*n*)		671	460	405	343
		**Questionnaires matching count of answers (*****n*****)**			
Answers given (*n*)	4	133	–	–	–
	3	40	115	70	58
	2	10	47	72	55
	1	4	21	51	59
	0	5	9	2	20

*Respondents were not required to answer all provided free text boxes to continue the questionnaire. n, number*.

Asked for their believe in regard to the average time likely passing from symptom onset to confirmation of childhood stroke the participants answered as follows: *time slot earlier than 1 h* was ticked once, *time slot 1–6 h* 36 times (19%), *time slot 7–12 h* 41 times (21%), *time slot 13–24 h* 42 times (22%), *time slot 25–48 h* 36 times (19%), and *time slot later than 48 h* 36 times (19%).

### Former childhood stroke specific training (multiple-option question)

Regarding former childhood stroke specific training, *participation in advanced educational sessions* was stated in 74 (39%) and *study of specific literature* in 118 questionnaires (61%) Other, previously preferred sources of information about childhood stroke included *discussion with colleagues in 71 (37%)* and *internet research* in 48 cases (25%).

### Demand for future stroke specific skill enhancement (multiple-option question)

**Figure 4:**
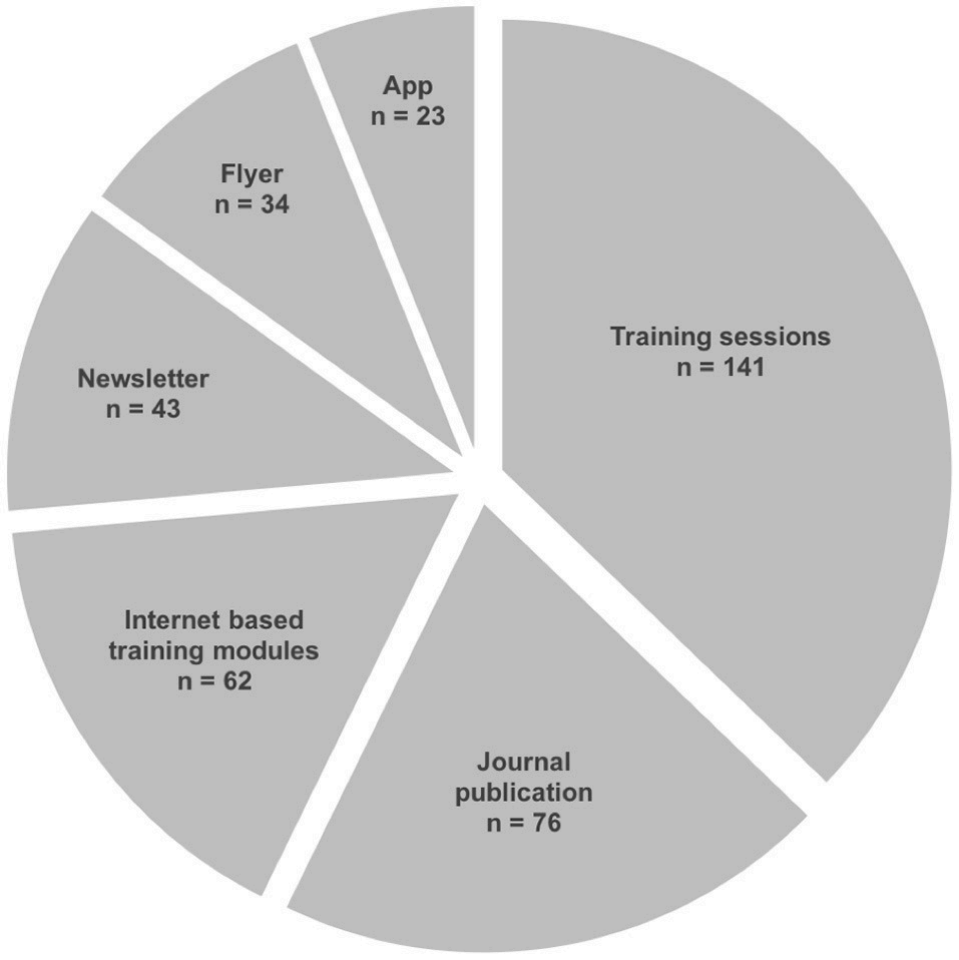
Demand for future stroke specific skill enhancement. Physicians were asked about their preferred method of future stroke specific skill enhancement by offering the depicted six items as multiple choice options. Physicians could choose as many options as they considered important. N, number.

In 178 questionnaires (93%) an interest in personal skill enhancement on the topic of childhood stroke was stated. Of the given multiple-choice options, the most preferred method was *participation in advanced training sessions* (*n* = *141, 80*%). *Journal publications, internet-based training modules, newsletter, flyer*, or *training app* were appreciated in 76 (43%), 62 (35%), 43 (24%), 34 (19%), and 23 (13%) cases, respectively (Figure 4). Consent for offering a flyer on the topic to caregivers in the institution was given in 94 (49%) questionnaires.

## Discussion

Expert opinion postulates low awareness of childhood stroke in the professional community and the public to be one of the main obstacles to optimize the stroke care. Consequently, the need for educational interventions to substantially improve early stroke recognition has been emphasized (5, 7, 8, 18). To our knowledge, no trial has specifically assessed professional or public awareness of childhood stroke so far.

We conducted this currently unique survey to capture a snap-shot of the awareness of, the interest in and the knowledge of childhood stroke among a broad cohort of physicians practicing in pediatric doctor's offices, children's hospitals, SPZs, and a neuropediatric rehabilitation facility in Bavaria, Germany.

Our non-rewarded survey reached an overall return rate of 14% with a complete response rate of 11%. Generally, the literature reports low return rates for (1) email/web based surveys and (2) in the community of physicians, in particular of pediatricians (19–22). Given today's physicians' survey burden the authors consider a 11% return rate a satisfying response level, at least providing insight into trends. However, due to the limited sample of responses, findings cannot be rated as representative. Interestingly, even a topic as important in acute pediatric care as stroke does not catch more attention, despite the high accessibility and the anonymous, online nature of the survey. In regard of the level of professional training substantially more pediatricians responded to the survey than residents. The return rate was especially low in the cohort of pediatricians practicing in doctor's offices (5.5%), although they represent the first-line providers for families. The return rate for pediatricians practicing in institutions in the region of Munich was substantially higher (28%), which to the one hand may reflect the higher likelihood to be exposed to children with suspected childhood stroke in the hospital than in the doctor's office. On the other hand, these respond rates may reflect some success of the efforts to optimize stroke care for children and adolescents in this region. Due to the implementation of the first German Pediatric Stroke Unit in Munich, together with the regular meetings held by the *German Network Pediatric Stroke* the topic has been highlighted considerably in Munich since 2014. Future efforts should focus on how to capture the attention of pediatricians in doctor's offices and of residents in regards to a topic as time critical as stroke.

Our results endorse the clinical importance of the topic in all settings. Almost half of the responding physicians actively consider childhood stroke as differential diagnosis in their daily routine and provide care for patients who have previously experienced childhood stroke, respectively.

Overall, this survey reveals some considerable professional knowledge concerning the topic. Important symptoms associated with childhood stroke, relevant differential diagnoses, proper diagnostic steps to confirm diagnosis, and adequate treatment options have been quoted to the free text questions by our cohort (23–26). However, three remarks have to be considered in rating these results: (1) The free text question type chosen offers better insight into knowledge than multiple choice questions. However, it was not required to fill all text boxes provided to continue the questionnaire. Therefore, number of answers given in relation to maximum answers possible has to be taken into account. In this survey proportion of given answers declines continuously the more specific the content gets. Further, we cannot differentiate if the answers are based on (2) a general knowledge of adult stroke or (3) general pediatric knowledge and experience rather than a specific overall comprehension of childhood stroke. Rating seizure as an important symptom of stroke could point at the latter as adult stroke is only very rarely presenting with seizure. On the other hand, seizure as a symptom of childhood stroke tends to be overemphasized in our survey as is impairment of consciousness. Hemiparesis and speech disorder are appropriately regarded as significant symptoms whereas the importance of facial palsy is underestimated in our sample (25, 27, 28).

Likewise, due to the less specific presentation and an age-dependent spectrum the number of stroke mimicking conditions in childhood is by far more extensive than in adulthood. Thus, the range of differential diagnoses given in our survey could reflect specific knowledge of this issue. However, the answers tend to display general neuropediatric entities rather than selected differential diagnoses of acute focal neurological dysfunction with rating brain infection and brain tumor within the top four stroke mimics. Therefore, relevant childhood stroke mimics as Bell's palsy, postictal paresis and somatoform disorders tend to be underestimated by our cohort (23, 27, 29).

In terms of proper diagnostic modalities to confirm a childhood stroke, our cohort is aware of the need for acute neuroimaging. MRI with MR-angiogram represents the gold-standard in children and has been quoted by the vast majority of participants. However, CT-scanning has been indicated by a relevant number of respondents, too. This result again may reflect a more adult stroke than specific childhood stroke approach. Due to the by far higher probability of stroke in adults presenting with acute onset focal symptoms, CT-scanning in the early stage serves to exclude hemorrhage, which represents a contraindication for thrombolysis. Contrarily, in line with recently published recommendations, CT is the modality of choice in children in critical condition, with preexisting contraindications to MRI, in need for sedation for MRI delaying diagnostics or presenting to a center without MRI capability, only. So far, we have not assessed if the physicians are aware of these recommendations and the limited sensitivity of head CT, in particular during the first 6 h (30). The other neuroimaging methods cited by our sample (transfontanellar ultrasound, conventional catheter angiogram, sonography of vessels) do not play a major, reliable role during acute childhood stroke work up (25, 30). Some answers pointed at the diagnostic work up to elucidate the etiology and evaluate common risk factors. This work up is important in the means of offering proper acute and long-term treatment and reducing the risk of recurrence. However, this work-up usually takes place in the post-acute management period after the stroke has been neuro-radiologically confirmed and was not target of this question.

Regarding the acute therapeutic measures, the knowledge gap appears to be especially important. Up to 80% of participants consider *thrombolysis* a first-line option whereas only about 40% indicate *anticoagulation* and even less (15%) *neuroprotective measures*. In contrast to adulthood, pediatric stroke patients are far less often eligible for thrombolysis, but anticoagulation is indicated in a majority of children suffering acute stroke and neuroprotective measures should be taken in any case (18, 25, 31). Nevertheless, for those patients to whom a thrombolysis could be associated to a major benefit in regards of long-time outcome we complaisantly emphasize that this option is being taken into account by that many responders. Evidently the specific treatment of children hit by stroke should take place in a specialized, multidisciplinary center experienced in this field. However basic knowledge on the options should be made available to any pediatrician to ensure initiation of basic neuroprotective measures and contacting to appropriate comprehensive centers.

In summary, even in a group of physicians, of whom the majority is well-trained in pediatrics and in whom a substantial proportion obviously are aware of and interested in the topic further knowledge improvement should be pursued.

In this context, the stroke mnemonic FAST is not known to the majority of the responding physicians, or cannot be explained properly. The rate of FAST knowledge is particularly low in the respondents practicing in doctor's offices although those physicians represent the first-line health care providers to families. FAST represents a well-established adult stroke screening tool, which promotes early recognition and fast tracks proper diagnostic pathways (32, 33). The low recall rate of FAST is particularly interesting given its importance in neurology and public stroke awareness campaigns throughout the world. However, it may reflect the lack of a sustained nation-wide FAST campaign in Germany. Due to its feasibility, FAST can serve as a basic triage tool in children presenting with acute brain attack, even though it has been shown to not be as reliable in children as in adults (34–36). Some educational programs on adult stroke have launched the new mnemonic BE FAST—prefixing B for “balance” and E for “eyes.” A recently published retrospective analysis revealed BE FAST to be more sensitive than FAST in detecting adult acute ischemic stroke (37). Given the high discriminative power of the symptom of leg weakness in regard to differentiating childhood stroke and stroke mimics, and the higher proportion of posterior circulation stroke in childhood, a positive impact of BE FAST on recognition rates could be assumed, also (27). Moreover, promoting BE FAST seems promising, because this acronym implicates not only the cardinal symptoms of stroke, but also the call to act promptly. Therefore, it may reach a higher sustainability rate than FAST alone, and in our opinion, should be used in future pediatric and adult stroke campaigns in preference to FAST.

Any kind of former training sessions on the topic of childhood stroke have been attended by at least 40% of respondents. This proportion is particularly high in the subgroup of physicians correctly recalling FAST (40 out of 52 physicians). Regarding future professional skill enhancement, the interest in training sessions is remarkably high. On behalf of the *German Network Pediatric Stroke*, regular meetings concerning the topic of pediatric stroke have been established throughout Germany since 2014. These meetings offer a major platform for exchanging experiences, an opportunity for addressing specific issues between groups of specialists, and educational lectures.

The results of this survey support the expert opinion that upcoming and advanced sessions for pediatricians and emergency care providers should focus on triage in the acute setting, and further diagnostic and management pathways (7, 17, 18). When developing such training modules, the difference between knowledge and awareness should be kept in mind. Whereas knowledge can be sufficient, actively taking acute stroke into account as a differential diagnosis in a pediatric cohort may still present a major barrier, due to limited awareness. Therefore, skill enhancement should be embedded in medical studies and take place on a regular basis, to keep the topic of strokes in children present in pediatricians' minds. Besides educational lectures, hands-on modules could enhance skills. Simulated training settings allow for becoming familiar with handling the situation of an acute stroke, and may contribute to persistently raising awareness of a rare but clinically impactful incident like childhood stroke.

Journal publications and the internet represent another well-accepted, existing, and future source of information for the cohort of responding physicians. A website conceived to offer general and advanced information on the topic could be an efficient approach. Training sessions in terms of webinars and an embedded expert forum could complement the range of content. In the last years, a fairly broad variety of Anglo-American websites has been launched, addressing professionals and the public, whereas the European and in particular the German-language web based offers are limited (10). No matter the type of the intervention, or what target group it is designed for, the evaluation of its sustainability is pivotal. The primary objective of this evaluation should be measuring the knowledge retention over time (38).

If professional awareness of the topic is limited, public awareness can be assumed to be even lower. In fact, the issue of childhood stroke has not yet been introduced comprehensively to the public, particularly not in Europe. Public multi-media campaigns for adult stroke awareness have led to earlier stroke recognition, better outcomes, and decreased stroke-related health-care costs (38–42). Given the low a priori probability of acute childhood strokes, embedding the topic into successful existing adult stroke campaigns should be considered. A broadened campaign could cover general stroke information, from the newborn period up to seniority, highlighting the key message: *Be fast*—*adequate management of stroke requires correct diagnosis as early as possible-at any age*. This approach appears to have the potential to effectively bridge gaps in public childhood stroke awareness, without inappropriately worrying the parents. As a first and cost-efficient step, an informative flyer could be displayed in pediatric health care institutions, a measure appreciated by the half of the responding physicians.

To what extent this survey itself has contributed to raise the awareness of childhood stroke in the cohort of the invited and participating physicians is not traceable. However, spreading its results to the community of pediatricians could raise the awareness of the topic. If so, one more step has been taken to further optimize the care of children suffering stroke in line with the Global Stroke Bill of Rights of the World Stroke Association (43).

There are some limitations to our study: Technically, repeated completion by one and the same physician could not be excluded. Furthermore, the online character offers the possibility for parallel (internet) enquiry on the topic. About 20% of questionnaires were incompletely recaptured. Difficulty answering the questions or missing the relevance of the topic may have been reasons. The latter could even have deterred from starting the survey in the first place. Particularly in regards to the low response rate selection bias should be considered. Physicians with a particular interest in the topic or recent exposure to a patient with childhood stroke may have been more likely to complete the survey. This could have promoted overly positive results. Therefore, our findings cannot be rated as representative. The results of this trial may not reflect the awareness, relevance, knowledge and interest in the issue throughout other regions of Germany or Europe.

In the future, subsequent surveys could address physicians in other regions of Germany or Europe to display regional differences and common interests to define future directions in childhood stroke clinical practice and research. Moreover, further efforts should involve first-line responders (i.e., paramedics, nurses practicing in pediatric emergency departments). This group of medical professionals tends to be as much or even more likely than physicians to be in a position to rapidly triage potential childhood stroke patients. Therefore, their awareness of the topic is invaluably important.

Based on the experience attained during this trial the questionnaire should be refined to further enhance the significance of findings of subsequent studies. To assess the likelihood of selection bias through recent exposure a question as to *when the last time the participant either encountered a childhood stroke or received some formal education about the topic* would be reasonable. Selection bias due to a particular interest in the topic could be assessed by a question as to *if the participant engages in a comprehensive childhood stroke center, clinical care pathway, research team and/or a pediatric stroke network*. Regarding clinical importance of childhood stroke in daily routine a denominator should be recorded to improve interpretation of the given numbers (i.e., number of children seen for neurological reasons during the last 12 months).

Further, some technical enhancements could be implemented for future surveys on childhood stroke. The survey validation (“piloting”) should rather be conducted underneath a representative sample of professionals of the target group than of members of the expert panel. As to raise the response rate some actions to improve reporting should be discussed (i.e., individual personalized email invitation, timing of reminders, follow-up telephone call, duration of accessibility, monetary, or gift incentives) (19, 20).

## Conclusion

Childhood stroke constitutes a topic of clinical importance to pediatricians in daily professional routine in all settings. The participants reveal a considerable comprehension concerning the subject. Nevertheless, given an event as time-critical as acute stroke, there still remains substantial room for improvement. A multi-modal approach to raise awareness and knowledge of childhood stroke could be comprised of the following educational interventions: (1) The implementation of a training program composed of lectures and hands-on training, covering triage and the course of emergency diagnostics and treatment. These sessions could target the whole group of (pediatric) health care providers, including medical students, nurses, paramedics, general practitioners, and pediatricians. Regular evaluation of the impact of these sessions would allow for continuous enhancement to the program. (2) Via regular publications in professional journals and an elaborate website on the topic, an even broader range of physicians could be reached. Further, an European/German public awareness campaign based on the BE FAST mnemonic, covering the key message: “*Strokes can occur at any age, from 0 to 99 years old!”* could complement these interventions. All of these efforts will contribute to optimize the care of children suffering from stroke, in line with the Global Stroke Bill of Rights of the World Stroke Association.

## Ethics statement

The study was approved by the ethics committee and data protection commissioner of the medical faculty of the Ludwig-Maximilians-University Munich, Germany, Project-Nr. 455–16. The data presented in this manuscript are the results of analysis of the responses to an anonymous online survey sent out to pediatricians in Bavaria, Germany. The survey contained a note regarding the protection of data privacy. This note declared that no personal or individual-related data were to be collected during the completion of the survey. This information on the anonymous data-management had to be confirmed to begin completing the questionnaire on the topic of childhood stroke. The ethics statement is uploaded as Supplementary Material, named Data Sheet 2.

## Author contributions

LG, JG, MB, KB, and RW conceptualized and designed the study, contributed to the acquisition, analysis and interpretation of data. LG, MB, and KB drafted the initial manuscript. IB, FH, SS, MO, ML, KV, MT, SB, KR, FH, and TN contributed to the interpretation of the data and reviewed and revised the manuscript. All authors reviewed and approved the final manuscript as submitted.

### Conflict of interest statement

The authors declare that the research was conducted in the absence of any commercial or financial relationships that could be construed as a potential conflict of interest. The reviewer RF and handling Editor declared their shared affiliation.
