# Supplementary material for: Childhood Stroke: Awareness, Interest, and Knowledge Among the Pediatric Community
Source: Front Pediatr. 2018 Jun 25;6:182. doi: 10.3389/fped.2018.00182 (PMC6026646; doi:10.3389/fped.2018.00182)
Supplement: Supplementary file 1 [file Data_Sheet_1.PDF]

Supplementary Material

Childhood stroke: Awareness, interest and knowledge among the pediatric community

Short title: Awareness of childhood stroke

Bonfert MV, Badura K, Gerstl J, Borggraefe I, Heinen F, Schroeder AS, Olivieri M, Weinberger R, Landgraf MN, Vill K, Tacke M, Berweck S, Reiter K, Hoffmann F, Nicolai T, Gerstl L*

* Corresponding author

E-mail: lucia.gerstl@med.uni-muenchen.de (LG)

Survey on the awareness of childhood stroke

Questionnaire

1. **How often have you been considering a childhood stroke as diagnosis during the past 12 months?**

   Please choose one of the following options:

   - ○ 0 times
   - ○ 1-5 times
   - ○ >5 times

2. **Do you follow up children who have suffered childhood stroke?**

   Please choose one of the following options:

   - ○ Yes
   - ○ No

3. **Have you heard of the mnemonic FAST?**

   Please choose one of the following options:

   - ○ Yes
   - ○ Yes, but I do not recall its meaning
   - ○ No

4. **If you have answered “Yes” to question 3, please quote the meaning of the mnemonic FAST:**

   - **F**
   - **A**
   - **S**
   - **T**

5. **Please list here up to 4 symptoms of a childhood stroke:**

   -
   -
   -
   -

6. **Please list here up to 3 mimics of a childhood stroke:**

   -
   -
   -

7. **What kind of diagnostic modality contributes to confirm the diagnosis of childhood stroke (list up to 3 modalities):**

   -
   -
   -

8. **What kind of treatment options for acute intervention in case of childhood stroke do you know? Please list up to 3 options and their corresponding time frame from symptom onset to therapy:**

   -
   -
   -

9. Usually, after how many hours the diagnosis of childhood stroke will be confirmed on average?

Please choose one of the following options:

- ○ <1 hour
- ○ 1-6 hours
- ○ 7-12 hours
- ○ 13-24 hours
- ○ 25-48 hours
- ○ >48 hours

10. Are you interested in participating in an educational intervention on the topic of childhood stroke?

Please choose one of the following options:

- ○ Yes
- ○ No

11. What kind of educational interventions do you prefer?

Please choose all answers that apply:

- ☐ Flyer
- ☐ Training sessions
- ☐ Internet based training modules
- ☐ App
- ☐ Newsletter
- ☐ Journal publication

12. Have you previously been participating in training sessions on the topic of pediatric stroke?

Please choose one of the following options:

- ○ Yes
- ○ No

13. Have you previously been reading journal publications on the topic of pediatric stroke?

Please choose all answers that apply:

- ☐ No
- ☐ Yes, in the journal *„Monatsschrift Kinderheilkunde“*
- ☐ Yes, in the journal *„Kinder- und Jugendarzt“*
- ☐ Yes, publications in other journals

14. Have you been using other sources of information?

Please choose all answers that apply:

- ☐ No
- ☐ Yes, via television
- ☐ Yes, via internet
- ☐ Yes, via support groups
- ☐ Yes, via colleagues

15. Would you agree to dispose some flyer on the topic of childhood stroke addressing care-givers in your facility?

Please choose one of the following options:

- ○ Yes
- ○ No

16. In what kind of facility do you practice at?

Please choose one of the following answers:

- ○ Children’s hospital
- ○ Doctor’s office
- ○ Social pediatric center (SPZ)
- ○ Neuropediatric rehabilitation facility

17. My occupational title is:

Please choose one of the following answers:

- ○ Resident in pediatrics
- ○ Pediatrician
- ○ Pediatrician with further specialization

18. If you have chosen "pediatrician with further specialization", please choose all that apply:

- ○ Neuropediatrics
- ○ Pediatric oncology and hematology
- ○ Pediatric cardiology
- ○ Neonatology
- ○ Other: please quote here: ____
