# Supplementary material for: Childhood Stroke: Awareness, Interest, and Knowledge Among the Pediatric Community
Source: Front Pediatr. 2018 Jun 25;6:182. doi: 10.3389/fped.2018.00182 (PMC6026646; doi:10.3389/fped.2018.00182)
Supplement: Supplementary file 2 [file Data_Sheet_2.PDF]

Ethikkommission ·Pettenkoferstr. 8 · 80336 München

Frau
Dr. Lucia Gerstl
Dr. von Haunersches Kinderspital
Lindwurmstraße 4
80337 München

Vorsitzender:
Prof. Dr. W. Eisenmenger
Telefon+49 (0)89 440055191
Telefax+49 (0)89 440055192
Ethikkommission@
med.uni-muenchen.de
www.ethikkommission.med.uni
-muenchen.de

Anschrift:
Pettenkoferstr. 8a
D-80336 München

17.08.2016/sc

Projekt Nr: **455-16** (bitte bei Schriftwechsel angeben)

Beratung nach Fakultätsrecht

Studientitel:	Awareness des kindlichen Schlaganfalls bei Kinderärzten - eine fragebogengestützte Online-Umfrage im Raum München/Deutschland
Antragsteller:	Dr. Lucia Gerstl, Prof. Dr. Florian Heinen, Dr. von Haunersches Kinderspital, Lindwurmstraße 4, 80337 München

Sehr geehrte Frau Dr. Gerstl,

die Ethikkommission hat Ihren Antrag zur o.g. Studie auf der Basis der vorgelegten Unterlagen und Informationen geprüft.

Die Ethikkommission (EK) erhebt keine Einwände gegen die Durchführung der Studie.

Allgemeine Hinweise:

- Änderungen im Verlauf der Studie sind der EK zur erneuten Prüfung vorzulegen.
- Schwerwiegende unerwartete Ereignisse im Rahmen der Studie sind der EK mitzuteilen.
- Das Ende der Studie ist anzuzeigen und das Ergebnis vorzulegen.
- Die ärztliche und juristische Verantwortung bei der Durchführung der Studie verbleibt uneingeschränkt bei Ihnen und Ihren Mitarbeitern.

Die Ethikkommission wünscht Ihnen für Ihr Vorhaben viel Erfolg.

Mit freundlichen Grüßen

Prof. Dr. W. Eisenmenger
Vorsitzender der Ethikkommission
